# Effects of the COVID-19 Epidemic on Hospital Admissions for Non-Communicable Diseases in a Large Italian University-Hospital: A Descriptive Case-Series Study

**DOI:** 10.3390/jcm10040880

**Published:** 2021-02-21

**Authors:** Caterina Caminiti, Giuseppe Maglietta, Tiziana Meschi, Andrea Ticinesi, Mario Silva, Nicola Sverzellati

**Affiliations:** 1 Research and Innovation Unit, University Hospital of Parma, 43126 Parma, Italy; ccaminiti@ao.pr.it; 2 Geriatric-Rehabilitation Department, University Hospital of Parma, 43126 Parma, Italy; tiziana.meschi@unipr.it (T.M.); aticinesi@ao.pr.it (A.T.); 3 Radiological Sciences Unit, University Hospital of Parma, 43126 Parma, Italy; mario.silva@unipr.it (M.S.); nsverzellati@ao.pr.it (N.S.)

**Keywords:** COVID-19, epidemiology, hospital admissions, non-communicable diseases, coronavirus infection

## Abstract

Background: Concern is growing about the negative consequences that response measures to the COVID-19 epidemic may have on the management of other medical conditions. Methods: A retrospective descriptive case-series study conducted at a large University-hospital in northern Italy, an area severely hit by the epidemic. Results: Between 23 February and 14 May 2020, 4160 (52%) COVID-19 and 3778 (48%) non-COVID-19 patients were hospitalized. COVID-19 admissions peaked in the second half of March, a period characterized by an extremely high mortality rate (27.4%). The number of admissions in 2020 was similar to 2019, but COVID-19 patients gradually occupied all available beds. Comparison between COVID-19 and non-COVID-19 admissions in 2020 revealed significant differences concerning all age classes and gender. Specifically, COVID-19 patients were older, predominantly male, and exhibited more comorbidities. Overall, admissions for non-communicable diseases (NCDs) in 2020 vs. 2019 dropped by approximately one third. Statistically significant reductions were observed for acute myocardial infarction (−78, −33.9%), cerebrovascular disease (−235, −41.5%), and cancer (−368, −31.9%). While the first two appeared equally distributed between COVID-19 and non-COVID-19 patients, chronic NCDs were statistically significantly more frequent in the former, except cancer, which was less frequent in COVID-19 patients. Conclusions: Prevention of collateral damage to patients with other diseases should be an integral part of epidemic response plans. Prospective cohort studies are needed to understand the long-term impact.

## 1. Introduction

The Coronavirus disease 2019 (COVID-19) outbreak, announced as a pandemic by the World Health Organization (WHO) on 11 March 2020, imposed considerable strain on the health care systems of countries around the world [1]. Hospitals and community services were overwhelmed by the rapidly increasing number of affected patients requiring urgent care, and by the need to limit disease spread and rapidly identify and isolate new cases. In response to this unprecedented crisis, most hospitals adopted extraordinary measures, including resource reallocation, ward repurposing, and work reorganization [2,3]. At the same time, many countries enforced restrictive provisions to counter the spread of infection, including lockdowns, the closing of schools and commercial activities, social distancing, and travel restrictions [4].

These interventions, guided by the absolute priority placed on epidemic response, however, caused frequent delays or interruptions to ordinary care, particularly for people affected by non-communicable diseases (NCDs) [5,6]. Such changes to routine care could potentially affect quality-adjusted life years, making the socio, clinical, and economic impact of NCDs even worse. There are almost 94 million persons aged 65 and over in the EU, accounting for an 18.5% share of the population, and the majority of persons in this age group have multimorbidity, defined as multiple concurrent NCDs [7]. Stressing the importance of these issues, some authors have spoken of a “twin epidemic” which acted synergistically by increasing morbidity and mortality [5,6].

Italy was the first European country to be hit by the COVID-19 epidemic. Since the first confirmed case identified on 20 February in Lombardy (the region of Milan) in northern Italy, the outbreak quickly spread nationwide, particularly in the north, severely affecting the National Health Service [8]. Hospitals had to enact timely organizational and structural changes to counter rapidly increasing admissions and long lengths of stay (LOS) of infected patients. These included the closure and repurposing of hospital wards, restriction of visitor hospital access, identification of external triage areas, dedicated patient transport and isolation pathways, and the cessation of elective surgery [9,10,11,12]. All this has been made more difficult following the progressive cut of the national budget in the last decade, which has led to a strong reduction in beds, placing Italy below the European average with 12.9 beds per 100,000 inhabitants, while Germany had 29.2 beds [13,14].

The University Hospital of Parma, located in the Emilia-Romagna region, was particularly involved in the health crisis, also because, being a referring center for intensive care, it also admitted many infected patients from neighboring areas. Here, a specific algorithm has been implemented to manage the flow of suspected cases, which included pre-triage and a specific pathway enabling timely COVID-19 screening and rapid admission of positive subjects to COVID-19-dedicated wards [15].

This work aims to describe the impact of the epidemic on hospitalizations, particularly of subjects with NCDs, in the context of the hospital model outlined above, by comparing data from the period of the COVID-19 outbreak with the equivalent period in 2019.

## 2. Materials and Methods

### 2.1. Study Design, Setting and Participants

Study data were anonymously extracted from the electronic hospital discharge forms (eHDFs), contained in the administrative databases of the Emilia Romagna Regional Health Trust [17], and included the following: age, sex, medical comorbidities, dates of admission and discharge, and discharge status (deceased, transferred to a different hospital or home, etc.).

EHDFs contain the main diagnosis and up to five secondary diagnoses (i.e., any conditions existing at admission or occurring during hospitalization which influence treatment or length of stay). Since the beginning of the epidemic, a field has been added where physicians are required to indicate whether the patient is being treated for COVID-19 infection. 

### 2.2. Data Collection

This is a retrospective, descriptive case-series study conducted at a single academic medical center. The University-Hospital of Parma is a 1044-bed facility with a catchment area of >400,000 inhabitants, the second largest hospital in the Emilia-Romagna Region. In 2019, it recorded a total of 45,540 hospital admissions (of which one fifth concerned people living outside the Parma Province), 115,500 Emergency Department admissions, and 3,960,000 ambulatory services [16]. 

This study concerns consecutive cases hospitalized at our institution from 23 February to 14 May in the years 2019 and 2020. Data were used to measure a secondary objective of an observational study performed at the University-Hospital of Parma, approved by the local Ethics Committee. 

### 2.3. Statistical Analysis 

Sample size was equal to the number of patients hospitalized during the study period without any a priori statistical calculation. Continuous variables are presented as median and interquartile ranges (IQRs) or mean and standard deviations. Categorical variables are expressed as absolute (number) and relative (percentage) of patients.

To compare categorical variables, we applied the chi-square test, the Mann–Whitney test for continuous variables expressed with median and IQRs, and the independent samples t-test in case of mean and standard deviation. Corrections for multiple testing were not applied since this is a descriptive study, without formal calculations for sample size or for statistical power, and all comparisons had only explorative purposes.

## 3. Results

**Figure 1:**
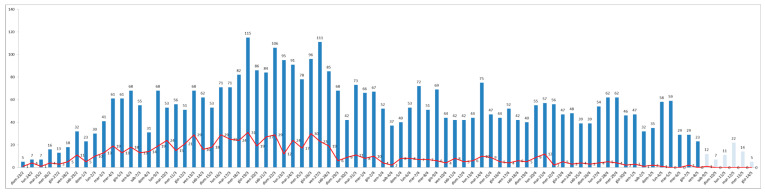
Trend of the number of COVID-19 admissions and corresponding deaths per day (red line).

Between 23 February and 14 May, 2020, there were 4160 (52%) COVID-19 and 3778 (48%) non-COVID-19 patients admitted to hospital. Figure 1 depicts the trend of the number of COVID-19 admissions and corresponding deaths per day (red line). COVID-19 admissions exhibited an increasing trend up to the second half of March, when the peak was reached, and then gradually decreased from April onwards. Concerning mortality, the death rate was extremely high (27.4%) since the beginning of the outbreak up to the end of March, and then gradually decreased (9.6%). 

**Figure 2:**
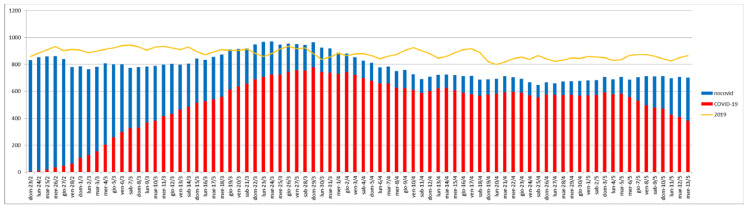
Number of patients present in the hospital each day during the epidemic period.

Figure 2 shows the number of patients present in the hospital each day during the epidemic in the period under consideration. Compared with the previous year (yellow line), the number of hospitalized patients during the epidemic did not increase, and the proportion of admissions for COVID-19 and non-COVID-19 patients was similar (4160 vs. 3778), though unequally distributed through time. In fact, as the days passed, COVID-19 patients gradually occupied nearly all available beds (red bars), with a corresponding reduction of non-COVID-19 patients (blue bars). 

Table 1 displays the comparison of characteristics between patients hospitalized during the epidemic and those admitted in the corresponding period of the previous year (7938 vs. 8656, −8.3%). Statistically significant differences were seen in age distribution: in 2020, admissions of individuals <18 years and between 40 and 64 years decreased, while those of people over 80 years increased. Considering admissions for the NCDs that are most frequently reported in the eHDF, in 2020, statistically significant reductions were observed for acute myocardial infarction (−78, −33.9%), cerebrovascular disease (−235, −41.5%), and cancer (−368, −31.9%). The comparison between COVID-19 and non-COVID-19 admissions in 2020 revealed significant differences concerning all age classes and genders. Specifically, COVID-19 patients were older, predominantly male, and exhibited more comorbidities. Regarding the frequency of NCDs, acute myocardial infarction and cerebrovascular disease appeared to be equally distributed between COVID-19 and non-COVID-19 patients, whereas chronic NCDs were statistically significantly more frequent in the former. Among chronic NCDs, cancer was the only exception, as it was less frequent in COVID-19 patients (7.8% vs. 12.1%). Regarding admissions for urgent surgical procedures, in 2020, a reduction was observed (−97, −37.7%), equally distributed between COVID-19 and non-COVID-19 patients.

Table 2 reports various process and outcome data. Concerning LOS, which in only 3% of cases was <24 h, the comparison between COVID-19 vs. non-COVID-19 patients in 2020 is particularly striking, as the value for the former was almost twice as high. This was probably due to the significantly larger proportion of elderly subjects (≥65-year-old patients made up 64.8% of COVID-19 vs. 32% of non-COVID-19 admissions), and to the greater number of comorbidities. The number of first admissions to intensive care in the two periods under consideration overlapped; however, in 2020 these predominantly consisted of COVID-19 patients. The analysis of patients who have had at least one access to intensive care shows that the percentage of patients in 2019 was almost equal to that of COVID-19 patients in 2020 (8.5% and 8.2%, respectively). Just as for overall LOS, LOS in intensive care was twice as long for COVID-19 patients as compared to non-COVID-19 patients in 2020, and to patients in 2019. Because of the disease severity and critical clinical conditions, the death rate among COVID-19 patients in 2020 was five times higher than the death rate in 2019 (19.2% vs. 4.1%).

Comparison of the outcomes between 2020 and 2019 for individual NCDs (Supplementary Table S1) confirmed an increase in the mortality rate also for non-COVID-19 patients, in particular for acute myocardial infarction, hypertension, and dementia, where the value was at least twice as high as in 2019. Concerning ICU use, in 2020, it was less frequent for all NCDs except for cancer, especially when associated with COVID-19 (8.9% vs. 5.5%).

## 4. Discussion

To our knowledge, this is the first study conducted in a large Italian public institution that provides a measure of the impact of the COVID-19 outbreak on NCD hospital admissions, compared with the corresponding period in 2019. In 2020, a decrease of nearly 10% in hospital admissions was observed, with different distributions by age group and a greater frequency of patients with comorbidities. This is in line with findings in the literature highlighting age and the presence of comorbidities as risk factors for hospitalization [18]. Overall, our data confirm the observations of a large study involving 201 US hospitals, where medical admissions in April 2020, the nadir period, declined by 34.1% [19]. Also, the decline in admissions for cerebrovascular disease and myocardial infarction is in line with experiences from all over the world. Among others, regarding coronary syndromes, a multicenter survey conducted on 54 Italian hospitals found a 48.4% reduction in admissions for AMI in one week during the COVID-19 outbreak compared with the equivalent week in 2019 (*p* < 0.001) [20]. Similarly, a nationwide retrospective survey on 17 Austrian centers conducted in March 2020 observed a significant decline in the number of hospital admissions due to acute coronary syndrome, reporting a relative reduction of 39.4% between the first and last calendar week [21]. As for strokes, according to a survey by the World Stroke Organization conducted across multiple countries, during the COVID-19 epidemic, the number of stroke admissions has fallen by as much as 50% [22].

Although the identification of the reasons leading to this phenomenon are beyond the scope of the present research, we speculate that a number of factors may have played a role. First, fear of becoming infected at the hospital may have discouraged people from seeking medical care, also following repeated invitations to reduce social contact issued by the government and the scientific community [23,24]. This may have been especially true in the case of milder symptoms, a hypothesis that seems to be supported by literature observations [20,25]. Interesting in this regard are the findings of Stuhr et al. [26], who found a 20% overall decline in cardiovascular admissions in January to April 2020 vs. the same period in 2019. The study highlights that this decline was mainly driven by a reduction in “discretionary admissions”, while “unavoidable admissions” were unchanged. These data may also lead us to speculate on the possibility that, in regular times (2019), admissions may be influenced to some degree by psychosomatic symptoms, to which people paid less attention during the outbreak.

Another possible reason for the reduction of NCD admissions may be related to a reallocation of healthcare resources during the pandemic, which may have caused less urgent problems to be deferred, possibly leading to a high rate of underdiagnosed diseases [24]. For instance, at our hospital, the CT scanner used for lung cancer secondary prevention was assigned to COVID-19 services, leading to temporary discontinuation of lung screening programs. Finally, we cannot exclude a paradoxical beneficial effect of social containment; more relaxed lifestyles during the lockdown, as well as the decrease of air pollution, may have contributed to a true reduction of some NCDs, such as myocardial infarction [27].

Our findings relating to cancer deserve specific consideration. It is noteworthy that the drop in cancer admissions in 2020 was mostly seen in COVID-19 compared to non-COVID-19 patients. We may speculate that this is suggestive of a lower incidence of COVID-19 in the oncology population, which could be explained by changes in clinical activity [28,29,30]. In fact, a survey conducted on the 12 medical oncology departments of the Emilia-Romagna region found that in 25% of centers the ward was temporarily closed or outpatient visits suspended, and that follow-up visits were canceled in two centers (16.7%), delayed in seven centers (58.3%), and performed by phone or remote assessment in seven centers (58.3%) [30]. We are not aware of other studies reporting reduced frequencies of COVID-19 admissions for cancer patients.

The analysis of mortality rates has shown a significant increase in 2020 for non-COVID-19 patients affected by some NCDs (acute myocardial infarction, hypertension, and dementia), a finding that deserves further investigation.

This study has some limitations. Firstly, data were taken from hospital administrative databases and were not collected prospectively for this research. However, the data quality is supposed to be similar in the two years we compared; thus, this aspect should not impact on its interpretation. Secondly, this is a single-center study; therefore, our results may have been influenced by local characteristics (disease incidence, local and regional health authority choice of restrictions, citizen behaviors and attitudes toward the epidemic, etc.). However, the similarities found with analogous research conducted in multiple countries suggests that our observations may be generalizable to other settings, and thus indicate the presence of a widespread problem. Finally, this study did not take into account reduced access to outpatient care, such as specialist consultations, tests, and screening programs, which may have had an even stronger impact on patients with NCDs.

### Future Implications

The analysis of what happened during the COVID-19 epidemic is invaluable to improve response and prepare for future crises of this kind. Various actions have already been taken at the local, regional, and national levels. 

One of the main lessons is the crucial role of community services and facilities and of their strong integration with specialized hospitals in ensuring continuity of care. Fortunately, the Emilia-Romagna region could benefit from well-established clinical networks based on a “hub and spoke” system. To ensure that citizens continued to receive appropriate care during the epidemic, with particular attention to vulnerable populations, since March 2020 the region has been issuing indications regarding specific areas (cardiology, oncology, hematology, etc.), addressed to health care professionals, defined by experts in accordance with existing guidelines and recommendations [31]. We also stress the need for educational initiatives addressed to citizens, aiming to both prevent COVID-19 infection and ensure proper management of NCDs [32]. Finally, this health emergency can be seen as an opportunity to improve and increase the integration of technology into routine patient care, such as the use of telemedicine and remote consultations. Such innovative approaches offer numerous potential advantages and are likely to be highly accepted by patients, for safety as well as logistical reasons, even after the COVID-19 crisis is over. Such potentials will be fully realized if the necessary communication infrastructure is made available to people wherever they live, and if the use of these services is ensured to all patients [33,34].

## 5. Conclusions

The consequences of the COVID-19 epidemic are not limited to patients affected by the virus. The prevention of collateral damage to patients with other diseases should be an integral part of any plan aiming to tackle such health crises. Attention to these aspects may also positively influence the outcome of vulnerable patients affected by COVID-19. Large prospective cohort studies of patients with NCDs are needed to fully understand the long-term impact of the current health crisis on these populations.

## Figures and Tables

**Table 1:** Baseline characteristics of hospitalized patients between 23 February and 14 May.

	Year 2019	Year 2020	*p*-Value	COVID-19 2020	Non-COVID-19 2020	*p*-Value
	(*n* = 8656)	(*n* = 7938)		(*n* = 4160)	(*n* = 3778)	
Age						
Median (IQR)	61 (33–78)	64 (35–79)	<0.001 ^Ɨ^	73 (59–82)	39 (11–71)	<0.001 ^Ɨ^
Class						
0	717 (8.3%)	831 (10.5%)	<0.001	44 (1.1%)	787 (20.8%)	<0.001
1–17	753 (8.7%)	356 (4.5%)	<0.001	132 (3.2%)	224 (5.9%)	<0.001
18–39	1171 (13.5%)	1075 (13.5%)	0.99	180 (4.3%)	895 (23.7%)	<0.001
40–64	2005 (23.2%)	1701 (21.4%)	0.007	1040 (25.0%)	661 (17.5%)	<0.001
65–80	2090 (24.1%)	1969 (24.8%)	0.67	1337 (32.1%)	632 (16.7%)	<0.001
80 +	1919 (22.2%)	1940 (24.4%)	<0.001	1361 (32.7%)	579 (15.3%)	<0.001
Sex						
F	4443 (51.3%)	4023 (50.7%)	0.41	1807 (43.4%)	2216 (58.7%)	<0.001
M	4212 (48.7%)	3915 (49.3%)	0.41	2353 (56.6%)	1562 (41.3%)	<0.001
Comorbidities						
Median (IQR)	2 (1–4)	4 (2–6)	<0.001 ^Ɨ^	4 (3–6)	2 (1–4)	<0.001 ^Ɨ^
Acute NCDs						
Acute Myocardial Infarction	230 (2.7%)	152 (1.9%)	0.002	73 (1.8%)	79 (2.1%)	0.32
Cerebrovascular Disease (Stroke, TIA)	566 (6.5%)	331 (4.2%)	<0.001	178 (4.3%)	153 (4.0%)	0.65
Chronic NCDs						
Hypertension	1420 (16.4%)	1360 (17.1%)	0.21	993 (23.9%)	367 (9.7%)	<0.001
Dementia	409 (4.7%)	328 (4.1%)	0.07	253 (6.1%)	75 (2.0%)	<0.001
Chronic Respiratory Disease (COPD, Asthma)	418 (4.8%)	334 (4.2%)	0.6	246 (5.9%)	88 (2.3%)	<0.001
Diabetes	751 (8.7%)	684 (8.6%)	0.9	495 (11.9%)	189 (5%)	<0.001
Cancer	1152 (13.3%)	784 (9.9%)	<0.001	326 (7.8%)	458 (12.1%)	<0.001
Urgent surgical procedures						
	257(3%)	160(2%)	<0.001	82 (2%)	78 (2.1%)	0.8

Ɨ *p*-value obtained by means of Mann–Whitney Test. No correction for multiple testing was applied. TIA, Transient Ischemic Attack.

**Table 2:** Admissions to intensive care unit (ICU), hospital stay and in-hospital mortality.

	Year 2019	Year 2020	*p*-value	COVID-19 2020	Non-COVID-19 2020	*p*-Value
	(*n* = 8656)	(*n* = 7938)		(*n* = 4160)	(*n* = 3778)	
LOS—days, Mean (SD)	8.3 (11.8)	7.6 (10.0)	<0.001 ^§^	9.9 (10.2)	5.2 (9.2)	<0.001 ^§^
First access to ICU	333 (3.8%)	246 (3.1%)	0.08	160 (3.8%)	86 (2.3%)	<0.001
Access to ICU at least once	737 (8.5%)	537 (6.8%)	<0.001	342 (8.2%)	195 (5.2%)	<0.001
LOS—days in ICU, Mean (SD)	5.2 (8.7)	7.9 (11.1)	<0.001 ^§^	9.9 (12.6)	4.4(5.9)	<0.001 ^§^
Deaths	351 (4.1%)	966 (12.2%)	<0.001	798 (19.2%)	168 (4.4%)	<0.001

^§^*p*-value obtained by means of independent samples *t*-test. No correction for multiple testing was applied. LOS, Lengths of Stay.

## Data Availability

The data presented in this study are available on request from the corresponding author. The data are not publicly available due to local privacy policy.

## Supplementary Materials

The following are available online at https://www.mdpi.com/2077-0383/10/4/880/s1, Table S1: Admissions to ICU and in-hospital mortality for individual NCDs.
