# Supplementary material for: Effects of the COVID-19 Epidemic on Hospital Admissions for Non-Communicable Diseases in a Large Italian University-Hospital: A Descriptive Case-Series Study
Source: J Clin Med. 2021 Feb 21;10(4):880. doi: 10.3390/jcm10040880 (PMC7924591; doi:10.3390/jcm10040880)
Supplement: Supplementary file 1 [file jcm-10-00880-s001.pdf]

Table S1. Admissions to ICU and in-hospital mortality for individual NCDs

	**Year 2019** (*n* = 8656)	**Year 2020** (*n* = 7938)	**Covid 2020** (*n* = 4160)	**non-Covid 2020** (*n* = 3778)
		**Access to ICU at least once**		
**Comorbidities**				
Acute NCDs				
Acute Myocardial Infarction	162/230 (70.4%)	101/152 (66.4%)	48/73 (65.7%)	53/79 (67.0%)
Cerebrovascular Disease (Stroke, TIA)	72/566 (12.7%)	24/331 (7.2%)	13/178 (7.3%)	11/153 (7.1%)
Chronic NCDs				
Hypertension	219/1420 (15.4%)	127/1360 (9.3%)	76/993 (7.7%)	51/367 (13.8%)
Dementia	4/409 (1%)	1/328 (0.3%)	1/253 (0.4%)	0/75 (0%)
Chronic Respiratory Disease (COPD, Asthma)	47/418 (11.2%)	25/334 (7.5%)	15/246 (6.1%)	10/88 (11.3%)
Diabetes	84/751 (11.1%)	63/684 (9.2%)	38/495 (7.7%)	25/189 (13.2%)
Cancer	63/1152 (5.5%)	57/784 (7.3%)	29/326 (8.9%)	28/458 (6.1%)
		Deaths		
Comorbidities				
Acute NCDs				
Acute Myocardial Infarction	12/230 (5.2%)	19/152 (12.5%)	11/73 (15.0%)	8/79 (10.1%)
Cerebrovascular Disease (Stroke, TIA)	64/566 (11.3%)	83/331 (25.0%)	62/178 (34.8%)	21/153 (13.7%)
Chronic NCDs				
Hypertension	63/1420 (4.4%)	330/1360 (24.2%)	288/993 (29.0%)	42/367 (11.4%)
Dementia	46/409 (11.2%)	98/328 (29.8%)	82/253 (32.4%)	16/75 (21.3%)
Chronic Respiratory Disease (COPD, Asthma)	33/418 (7.9%)	94/334 (28.1%)	84/246 (34.1%)	10/88 (11.3%)
Diabetes	47/751 (6.3%)	181/684 (26.4%)	162/495 (32.7%)	19/189 (10%)
Cancer	96/1152 (8.3%)	80/784 (10.2%)	54/326 (16.5%)	26/458 (5.7%)
